# Viability Analysis and High-Content Live-Cell Imaging for Drug Testing in Prostate Cancer Xenograft-Derived Organoids

**DOI:** 10.3390/cells12101377

**Published:** 2023-05-12

**Authors:** Annelies Van Hemelryk, Sigrun Erkens-Schulze, Lifani Lim, Corrina M. A. de Ridder, Debra C. Stuurman, Guido W. Jenster, Martin E. van Royen, Wytske M. van Weerden

**Affiliations:** 1 Department of Urology, Erasmus University Medical Center, Dr. Molewaterplein 40, 3015 GD Rotterdam, The Netherlands; a.vanhemelryk@erasmusmc.nl (A.V.H.); s.erkens-schulze@erasmusmc.nl (S.E.-S.); c.deridder@erasmusmc.nl (C.M.A.d.R.); d.stuurman@erasmusmc.nl (D.C.S.); g.jenster@erasmusmc.nl (G.W.J.); 2 Department of Pathology, Erasmus University Medical Center, Dr. Molewaterplein 40, 3015 GD Rotterdam, The Netherlands; m.vanroyen@erasmusmc.nl

**Keywords:** organoid, drug testing, viability assays, live-cell confocal microscopy, high-content screening, prostate cancer, patient-derived xenograft

## Abstract

Tumor organoids have been pushed forward as advanced model systems for in vitro oncology drug testing, with the eventual goal to direct personalized cancer treatments. However, drug testing efforts suffer from a large variation in experimental conditions for organoid culturing and organoid treatment. Moreover, most drug tests are restricted to whole-well viability as the sole read-out, thereby losing important information about key biological aspects that might be impacted due to the use of administered drugs. These bulk read-outs also discard potential inter-organoid heterogeneity in drug responses. To tackle these issues, we developed a systematic approach for processing organoids from prostate cancer (PCa) patient-derived xenografts (PDXs) for viability-based drug testing and identified essential conditions and quality checks for consistent results. In addition, we generated an imaging-based drug testing procedure using high-content fluorescence microscopy in living PCa organoids to detect various modalities of cell death. Individual organoids and cell nuclei in organoids were segmented and quantified using a dye combination of Hoechst 33342, propidium iodide and Caspase 3/7 Green, allowing the identification of cytostatic and cytotoxic treatment effects. Our procedures provide important insights into the mechanistic actions of tested drugs. Moreover, these methods can be adapted for tumor organoids originating from other cancer types to increase organoid-based drug test validity, and ultimately, accelerate clinical implementation.

## 1. Introduction

In vitro cancer research is progressively shifting from using traditional 2D cancer cell lines towards 3D organoid models. Tumor organoids have been established for many cancer types, either directly from patients (patient-derived organoids, PDOs) or from patient-derived xenografts (PDX-derived organoids, PDXOs), with the reliable preservation of genetic and molecular features of the originating tumor [1,2,3]. Tumor organoids have been widely employed to investigate organoid drug responses to standard-of-care treatments and their capability to recapitulate matching patient responses [1,3].

Overall, tumor organoids are expected to hold a lot of promise for personalized medicine approaches, with the potential of predicting clinical treatment efficacy [4,5,6,7,8,9]. However, recent prospective intervention trials showed conflicting results for organoid-guided treatment selection in individual patients [10,11]. One of the major restrictions for implementing tumor organoids in clinical decision making is imposed by the lack of methodological standards in organoid establishment and functional testing [12]. Experimental procedures vary among research groups, which hampers the robustness and reproducibility of drug testing results [13,14]. Secondly, most organoid drug screens use cell viability, which is extracted from intracellular ATP levels, as the main endpoint [13]. This bulk read-out does not provide insights on the underlying drug-induced effects and neglects the potential inter-organoid heterogeneity in drug responses. Microscopy, and more specifically live-cell imaging, is a promising tool to tackle these hurdles and investigate changes in (individual) organoid phenotype, growth rate and cell death in response to drug exposure [15,16,17,18].

In the current study, we provide an optimized procedure for the preparation of prostate cancer (PCa) PDXOs for drug testing purposes. We defined optimal drug testing conditions and essential quality control parameters when standard ATP-based viability assays are used. Next, we developed a high-content live-cell confocal imaging pipeline to investigate the drug-induced effects on tumor growth and cell death at the single-organoid level. Our methods can easily be adapted for PDOs and PDXOs originating from various cancer types. By applying easy-to-add fluorescent dyes, we provide an imaging protocol that allows rapid drug testing within a clinically relevant timeframe for PDO-based individualized drug response predictions. 

## 2. Materials and Methods

### 2.1. PDXO Culture

PDXOs were generated and cultured as described previously [3]. In short, 1000–1500 mm^3^ large PDX tumors were resected and dissociated using scissors, followed by enzymatic digestion: 1 h of collagenase A (2 mg/mL; Roche Diagnostics, Mannheim, Germany, cat. no. 11088793001) and 10 min of TrypLE Express (Thermo Fisher Scientific, Waltham, MA, USA, cat. no. 12605028), both at 37 °C. Digested tissue was passed through a 100 μm cell strainer (Falcon, Corning, NY, USA, cat. no. 352360) and resuspended in synthetic hydrogel (Noviogel-P5K, Noviocell, Sopachem, Ede, The Netherlands; medium–hydrogel ratio of 64.5:35.5). Thirty μL hydrogel domes were dispensed in 24-well plates (Costar, Corning, cat. no. 3527) and covered with culture medium. PC346C PDXOs were cultured in basic prostate growth medium (PGM basic [19], Supplementary Table S1) supplemented with 0.1 nM R1881 (synthetic androgen; Sigma-Aldrich, Saint Louis, MO, USA, cat. no. R0908) and 10 μM Y-27632 (ROCK-inhibitor; Adipogen, San Diego, CA, USA, cat. no. AG-CR1-3564-M025). PC2412 and PC2416-DEC PDXOs were cultured in adjusted prostate cancer organoid medium (APCOM [3,20], Supplementary Table S1), with 0.1 nM R1881 and 10 μM Y-27632 being standard medium components. The culture medium was refreshed twice a week. Brightfield images were acquired with a Nikon Eclipse TS2 microscope equipped with 4×, 10× CFI Achro brightfield objectives and a 20× Fluor ELWD objective, a DS-Fi3 camera and NIS-Elements imaging software (version 4.6; Minato, Tokyo, Japan).

### 2.2. PDXO Drug Testing

Depending on the originating PDX model, organoids formed after four to fourteen days of in vitro culture. Preassembled PDXOs were retrieved from the hydrogel domes by gently dissolving the thermo-sensitive hydrogel with ice-cold medium, and then passing them through a 100 μm cell strainer to eliminate large organoids and reduce size heterogeneity at the start of the experiment. Pooled PDXOs were carefully resuspended in Noviogel-P5K (medium-gel ratio 64.5:35.5) and seeded as eight μL hydrogel domes in 96-well plates, with an organoid density of 5000–10,000 per dome. Four days later, drug exposure was initiated: the culture medium was exchanged for medium containing the appropriate concentration of docetaxel (microtubule stabilizer; logarithmic dose range: 0.01–30 nM; Sanofi, Paris, France), cabazitaxel (microtubule stabilizer; logarithmic dose range: 0.01–30 nM; Sanofi), enzalutamide (anti-androgen; logarithmic dose range: 0.1–30 μM; Axon Medchem, Groningen, The Netherlands, cat. no. 1613), a vehicle (for negative controls) or staurosporine (broad-spectrum protein kinase inhibitor; 1 μM for positive controls; Selleckchem, Houston, TX, USA, cat. no. S1421). PDXOs were exposed for a total duration of 10 days, followed by viability assessment or confocal live-cell imaging.

### 2.3. Assessment of PDXO Viability

For viability-based drug testing, PDXOs were cultured in white clear-bottom 96-well plates (Costar, Corning, cat. no. 3610). During organoid seeding, two accompanying plates with from six to twelve replicate wells were prepared for PDXO viability assessment on day −4 (day of organoid seeding, four days before drug exposure) and day 0 (day of drug exposure initiation). Organoid viability was determined on day −4, day 0 and day 10 (end of drug exposure) using CellTiter-Glo 3D (Promega, Madison, WI, USA, cat. no. G9681) according to the manufacturer’s instructions. Preceding the standard instructions, plates were placed on ice for 30 min and shaken for 1 min at 800 rpm in order to dissolve the synthetic hydrogel without disrupting the organoid structure.

### 2.4. Confocal High-Content Live-Cell Imaging of PDXOs

For imaging-based drug testing, PDXOs were cultured in black 96-well CellCarrier Ultra plates (Perkin Elmer, Hamburg, Germany, cat. no. 6055302). One plate with twelve replicates was included to acquire baseline images at day 0. All treatment plates imaged on day 10 were provided with replicates exposed to 1 μM staurosporine six hours before image acquisition to serve as positive controls for cell death induction.

Fluorescent dyes were added to the wells three hours before imaging at a final concentration of 2 μg/mL for Hoechst 33342 (Invitrogen, Thermo Fisher Scientific, cat. no. H3570), 2 μM for Caspase 3/7 Green (CellEvent™, Invitrogen, Thermo Fisher Scientific, cat. no. C10723) and 1 μg/mL for propidium iodide (PI; HelloBio, Princeton, NJ, USA, cat. no. HB0820). Confocal imaging (spinning disk) was performed with the Opera Phenix High-Content Screening System (Perkin Elmer) equipped with a 40× water immersion objective (NA 1.1) and a 16-bit sCMOS 4 Megapixel camera, providing images with a field of view of 78,974.6 μm^2^. For each field of view, 15 planes were imaged with z-step sizes of 25 μm. Hoechst 33342, Caspase 3/7 Green and PI were excited with 405 nm, 488 nm and 561 nm solid state lasers and detected at 435–480 nm, 500–550 nm and 570–630 nm wavelength ranges, respectively. 

### 2.5. Image Analysis

A custom image analysis pipeline was developed with Harmony analysis software (version 4.9, Perkin Elmer). The sampling of organoids and cell nuclei throughout the dome was performed via image analysis on individual planes as follows. Organoids were segmented based on the combined image of the three imaging channels (Hoechst 33342, Caspase 3/7 Green and PI). Gaussian filtering and region resizing were conducted to eliminate debris-derived fluorescence in the organoids’ periphery. The number of organoids and mean organoid sizes (μm^2^) were quantified. Organoids with an area < 250 μm^2^ were excluded in order to eliminate single cells, small cell clumps and debris. Within the identified organoids, cell nuclei were segmented and quantified via nuclear Hoechst 33342 staining. Mean Caspase 3/7 Green and PI intensity were determined for each nucleus.

### 2.6. Data Analysis and Visualization

GraphPad Prism (version 9.5.0, GraphPad, San Diego, CA, USA) and Microsoft Excel (version 16.68) were used for data analysis and visualization. Schematic figures were created with Adobe Illustrator (version 24.3) and BioRender.com (accessed on 8 March 2023). Data were plotted as the mean with error bars of SD or SEM, as specified in the figure legends. *p* < 0.05 was considered to be statistically significant; Bonferroni correction was applied in case of multiple testing. Statistical tests are indicated in the figure legends.

The nonlinear regression curve fit method (log(inhibitor) vs. response with a variable slope and four parameters) was applied using GraphPad Prism to generate viability-based dose–response curves. 

Image-based cell death quantification was performed based on mean nuclear Caspase 3/7 Green and PI intensities; gating thresholds were determined on day 0 and day 10 untreated controls for each individual experiment (Supplementary Figure S1). We first identified the viable cell fraction based on the mean+2×SD of Caspase 3/7 Green and PI intensities in day 0 controls (Supplementary Figure S1A,B). Data points with intensities above both thresholds were considered to be dead cells. Within this dead cell fraction, we identified three cell populations: apoptotic cells (high Caspase 3/7 Green intensity and low PI intensity), necrotic cells (high PI and low Caspase 3/7 Green intensities) and cells in late apoptosis or secondary necrosis (high Caspase 3/7 Green and high PI intensities). Apoptotic and necrotic gating thresholds were determined on day 10 untreated controls and set as straight lines projected from the intersection of the viable cell Caspase 3/7 Green and PI thresholds at an angle to accommodate the observed curved intensity distributions (Supplementary Figure S1C–E). Data points between the apoptotic and necrotic gating thresholds were defined as cells in late apoptosis or secondary necrosis (Supplementary Figure S1F).

## 3. Results

### 3.1. Optimization of PDXO Drug Testing Conditions

**Figure 1:**
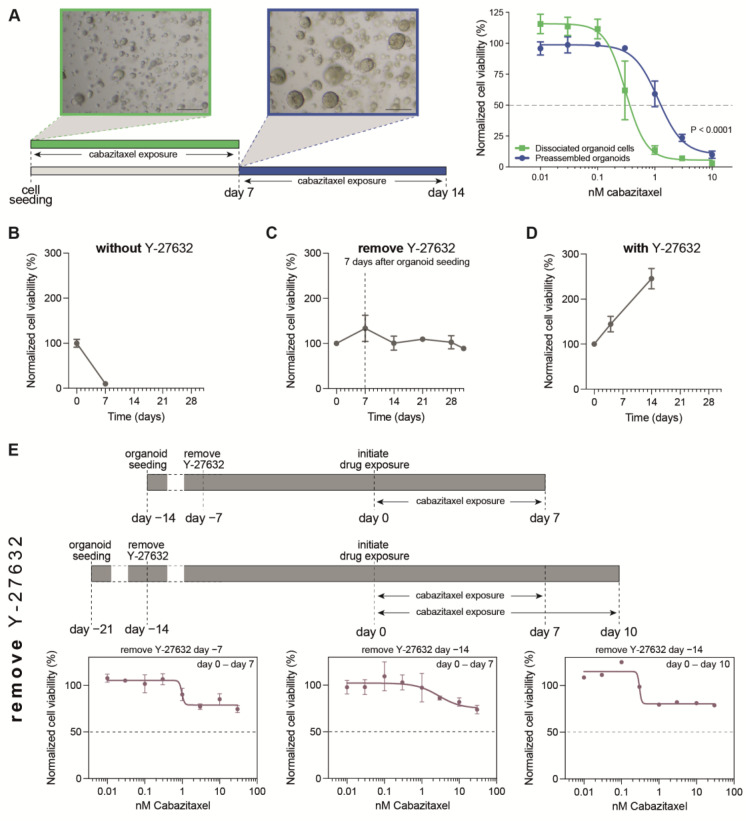

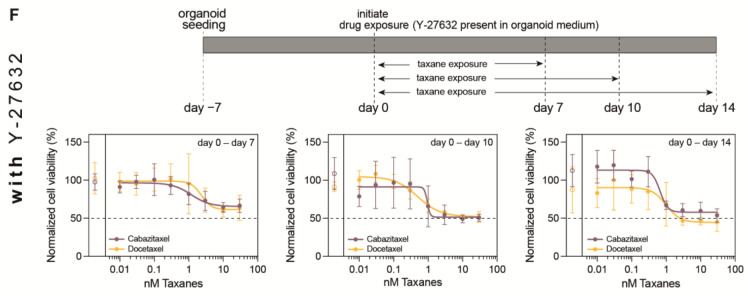
Optimization of PDXO drug testing conditions. (**A**) Impact of organoid formation prior to drug testing. Schematic representation of experimental design as applied in Van Hemelryk et al. [21]: drug exposure was initiated immediately after cell seeding, on dissociated organoid cells (green), or after 7 days, when organoids were formed (preassembled organoids, blue). Scale bar represents 100 μm. Cabazitaxel-exposed MSK-PCa1 organoids are shown as an example. Dose–response curves depict mean +/− SEM of four independent experiments with three technical replicates per dose, with viability normalized to ethanol controls. Data curated from [21]. (**B**–**D**) Impact of Y-27632 on organoid proliferation. Viability of PC2416-DEC PDXOs starting from the day of seeding, (**B**) without Y-27632 present in organoid culture medium, (**C**) with Y-27632 removed after 7 days, and (**D**) with Y-27632 continuously present in organoid culture medium. (**E**) Impact of organoid viability on treatment efficacy in correspondence with (**C**). Y-27632 was removed from culture medium 7 days after organoid seeding and from 7 to 14 days prior to drug exposure, as indicated on the timelines. PC2416-DEC PDXOs were exposed to cabazitaxel for 7 days (left and middle panel; mean +/− SEM of two independent experiments with six technical replicates per dose) or for 10 days (right panel; mean of six technical replicates per dose). Normalization to vehicle (ethanol) controls. (**F**) Impact of drug exposure time on treatment efficacy in correspondence with (**D**). In the presence of Y-27632, PC2416-DEC PDXOs were exposed to docetaxel or cabazitaxel for 7, 10 or 14 days (mean +/− SD of six technical replicates per condition). Normalization to vehicle (ethanol) controls of both treatment plates. Open dots represent vehicle controls of each treatment plate.

Previously, we demonstrated an impact of both the 3D organoid structure and the 3D culture environment on treatment efficacy in organoid-based drug tests [21]. Firstly, the sensitivity to standard-of-care treatments was markedly reduced in PCa organoids when the treatment started after organoid assembly as compared to that when 3D cultures of dissociated organoid cells were incubated prior to organoid assembly (Figure 1A). These findings underline the importance of organoid formation prior to drug exposure, to approximate the complex 3D organization of tumor cells within a solid tumor. Secondly, 3D scaffolds used for organoid culturing were found to delay and potentially attenuate drug efficacy. The use of a synthetic hydrogel (Noviogel-P5K), however, was associated with a scaffold-mediated delay of only a few hours (which can be considered as negligible in drug-exposure experiments spanning multiple days) and allowed to reach the same level of drug effect as that in the control conditions without the scaffold.

The abovementioned observations in established PCa organoid lines formed the basis for setting up a pipeline for drug testing in PCa PDXOs: drug exposure was initiated on preassembled organoids, cultured in the synthetic hydrogel Noviogel-P5K and in an optimized PDXO culture medium [3]. In addition, the current study investigated other potential confounders of drug efficacy that are specific to PCa PDXO cultures.

In established organoid lines, Y-27632 is generally added to the culture medium during the first days after seeding in order to prevent dissociation-mediated apoptosis [13,22]. Indeed, also in PCa PDXOs, we observed viability to collapse when seeding was performed without the presence of Y-27632 (Figure 1B). Discrepancies exist in adding Y-27632 to experimental medium for organoid drug testing. To prevent Y-27632 masking potential drug-induced apoptosis, we aimed to remove Y-27632 from our organoid medium seven days after seeding (and prior to drug testing), but we found short-term Y-27632 exposure to be incapable of sustaining PCa PDXO proliferation (e.g., PC2416-DEC, Figure 1C and PC2412, Supplementary Figure S2A). This is in strong contrast to uninterrupted Y-27632 exposure, which did maintain proliferation (Figure 1D). Performing drug tests in non-proliferative organoid cultures interferes with the correct interpretation of the drug’s effect, as was illustrated by an absent or minimal cabazitaxel effect in the drug tests where we removed Y-27632 from the organoid medium prior to treatment initiation (Figure 1E, Supplementary Figure S2B). Therefore, despite its potential counteracting effect on the tested drug, we have to conclude that Y-27632 is an essential component of the experimental medium when performing drug tests in PCa PDXO. 

Importantly, even in optimally proliferating PCa PDXO cultures (with continuous Y-27632 exposure), taxane efficacy was impaired when the drug exposure time was limited to 7 days (Figure 1F). Prolonged drug exposure was needed to reach an adequate level of taxane efficacy; however, this was achieved at the expense of higher variability between technical replicates and between treatment plates (Figure 1F, Supplementary Figure S3). This variability was most pronounced after 14 days of taxane exposure without an additional benefit for taxane efficacy, restricting the drug exposure time to 10 days.

### 3.2. Optimization of Viability Assays for Drug Testing in PCa PDXOs

**Figure 2:**
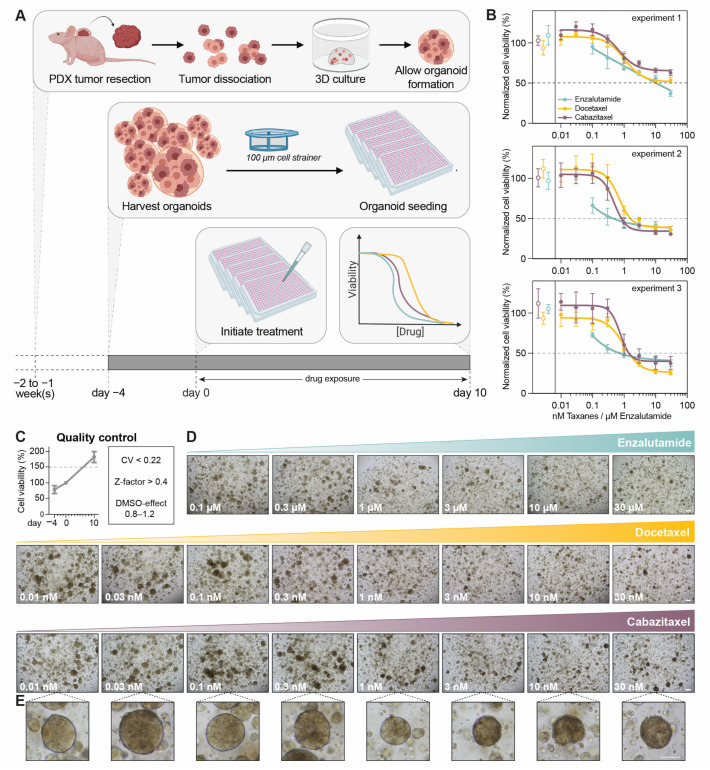
Optimization of PCa PDXO viability assays. (**A**) Schematic overview. (**B**) Reproducibility of the optimized procedure. Three independent experiments of PC2416-DEC PDXOs exposed to dose ranges of enzalutamide, docetaxel and cabazitaxel (mean +/− SD of six technical replicates per condition). Normalization to all untreated controls per experiment. Open dots represent untreated controls of each treatment plate. Data from [3], analyzed for individual experiments. (**C**) Quality control metrics of experiments in (**B**): i. increase in organoid viability of ≥ 1.5 between day 0 and day 10 (mean +/− SEM of three experiments; from 6 to 18 technical replicates for each timepoint); ii. coefficient of variation (CV) < 0.22 and Z-factor > 0.4 for each treatment plate; iii. DMSO effect between 0.8 and 1.2 for each enzalutamide treatment plate. (**D**) Representative brightfield images of PC2416-DEC PDXOs exposed to dose ranges of enzalutamide, docetaxel and cabazitaxel. Scale bar 100 μm. (**E**) Detail of a single organoid for each dose of cabazitaxel. Scale bar: 100 μm.

Taking into account the abovementioned identified confounders, we developed an optimized procedure for drug testing in PCa PDXOs (Figure 2A). The PCa PDX tumor of interest was resected, and PDXOs were generated as described above. Organoids were allowed to assemble for four to fourteen days, depending on the proliferation rate, after which preassembled PDXOs were harvested without disrupting the organoid structure. Pooled organoids were filtered to reduce size variation, and the organoid fraction of less than 100 μm in diameter was seeded in synthetic hydrogel domes. To ensure drug testing was performed during the proliferative stage, seeded PDXOs were allowed to recover for four days before drug exposure was initiated. During exposure for 10 days, PDXOs were incubated with a dose range of the drugs of interest. Negative and positive controls were included in each treatment plate. Y-27632 was continuously present in the organoid culture medium throughout the entire procedure. Finally, organoid viability was determined as the endpoint measurement of treatment efficacy. 

We demonstrated this optimized procedure to give highly reproducible results for replicate experiments in each PDX model exposed to standard-of-care therapeutics (Figure 2B, Supplementary Figure S4A). For the optimal interpretation of drug testing results, we endorse integrating a few quality control steps (Figure 2C*,* Supplementary Figure S4B). In some PDX models (e.g., PC2412, Supplementary Figure S5), we observed PDXO proliferation to be highly variable, even in the presence of Y-27632. To only include proliferating PDXOs, we applied a threshold of 150% for the organoid viability of untreated controls on day 10, as compared to the organoid viability before treatment initiation (day 0). In addition, standard quality control metrics, such as the coefficient of variation (CV), Z-factor and, if applicable, DMSO effect, were verified for each treatment plate [23]. In order to prevent the misinterpretation of drug testing results, we recommend excluding any treatment plate that does not meet these quality requirements.

Viability-based drug tests are restricted to (ATP-based) cell viability as the sole endpoint read-out. Although whole-well cell viability correlates with organoid load, it remains an indirect measurement and does not inform about the underlying drug-induced cellular response. A visual inspection after 10 days of drug exposure revealed smaller organoid sizes and a lower culture density with increasing dosages (Figure 2D, Supplementary Figure S4*C*), suggesting tumor growth inhibition occurred. However, the inspection of individual organoids also showed organoid dissociation (Figure 2E), indicating that additional biological processes such as cell death might be impacted by the administered drugs. These observations demanded for exploring imaging-based organoid drug tests.

### 3.3. Confocal High-Content Live-Cell Imaging for Drug Testing in PCa PDXOs

**Figure 3:**
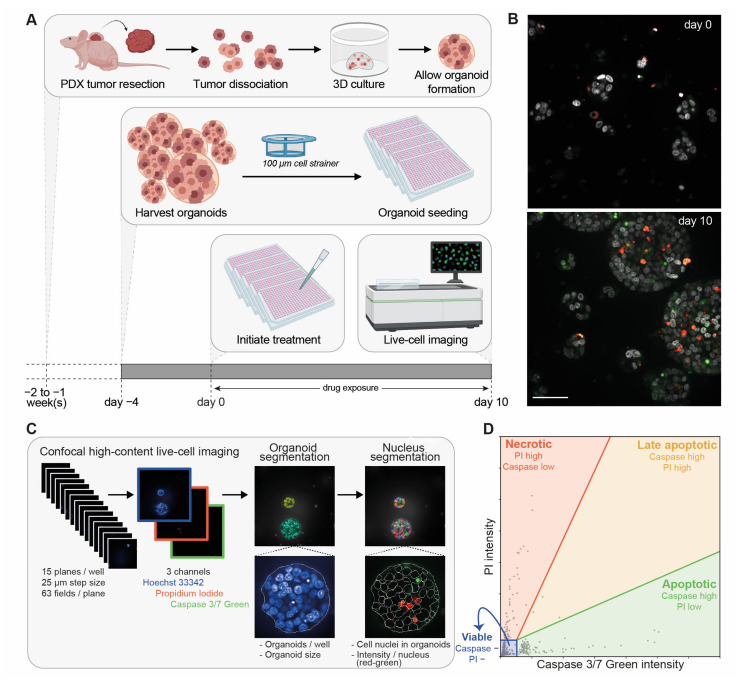
Optimization of confocal high-content live-cell imaging for drug testing in PCa PDXOs. (**A**) Schematic overview. (**B**) Representative confocal images of PC346C PDXOs stained with Hoechst 33342 (grey), Caspase 3/7 Green (green) and propidium iodide (red) before treatment initiation (day 0) and after drug exposure (day 10; untreated control). Images display the maximum projection of individual planes with step sizes of 25 μm. Scale bar represents 50 μm. (**C**) Schematic overview of our image analysis pipeline. Organoid segmentation is based on the combined image of all three fluorescent channels (Hoechst 33342, PI and Caspase 3/7 Green) and allows the quantification of the number of organoids per well and the mean organoid sizes (μm^2^). Within the segmented organoids, cell nuclei were identified and quantified based on nuclear Hoechst 33342 staining. Mean propidium iodide intensity (red) and Caspase 3/7 Green intensity (green) were determined per nucleus. (**D**) Schematic representation of image-based cell death identification using Caspase 3/7 Green intensity and propidium iodide (PI) intensity. Each data point represents one nucleus. Gating identified four cell populations: apoptotic cells (green; high Caspase 3/7 Green and low PI intensity), necrotic cells (red: high PI and low Caspase 3/7 Green intensity), late apoptotic cells (yellow: high Caspase 3/7 Green and high PI intensity) and viable cells (blue: negative (−) for both Caspase 3/7 Green and PI).

To allow the direct interpretation of drug effect on organoid load and cell death, we developed an optimized drug testing procedure using confocal high-content live-cell microscopy of PCa PDXOs. PCa PDXOs were cultured and processed as described above. After drug exposure for 10 days, living organoids were imaged instead of being lysed for viability determination (Figure 3A). 

PDXOs were stained with a dye combination of Hoechst 33342, PI and Caspase 3/7 Green (Figure 3B). We developed a custom image analysis pipeline for the segmentation and quantification of individual organoids and cell nuclei in organoids (Figure 3C). Within each identified nucleus, the fluorescent intensities of Caspase 3/7 Green and PI were measured. Caspase 3/7 Green is a membrane permeable dye that binds DNA once it is cleaved by caspase-3 or -7 in apoptotic cells. PI, on the other hand, can only enter the cell and bind DNA when the cell membrane has been compromised. Combining both dyes allows the capture of various modalities of cell death (Figure 3D): a high fluorescent intensity of Caspase 3/7 Green indicates apoptosis, a high intensity of PI indicates necrosis, and a high intensity of both dyes indicates membrane disruption during late apoptosis or secondary necrosis. The cell fraction without nuclear Caspase 3/7 Green or PI staining is considered to be viable. Single-dye control experiments excluded spectral crosstalk between both fluorescence channels (Supplementary Figure S6).

**Figure 4:**
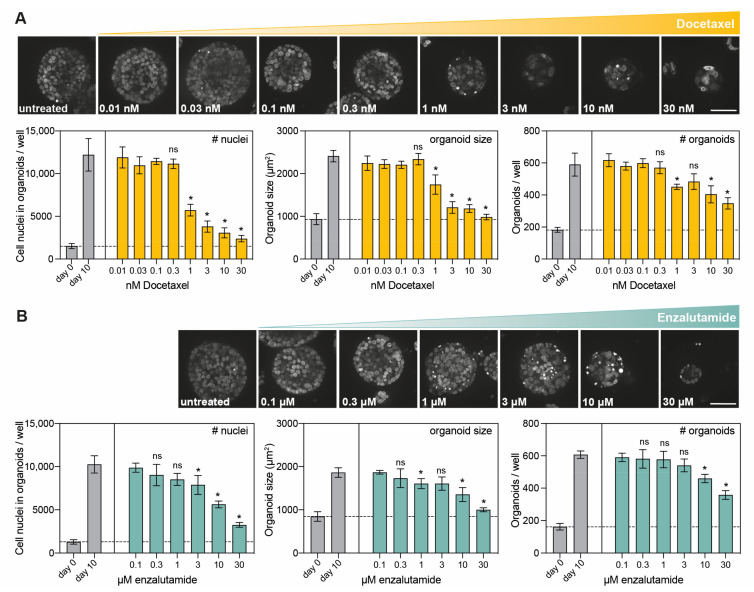
Image-based quantification of organoid load in PCa PDXOs exposed to standard-of-care therapeutics. (**A**) Representative confocal images (maximum projection) of Hoechst 33342-stained PC346C PDXOs exposed to a dose range of docetaxel. Scale bar: 50 μm. Image-based quantification of the total amount of cell nuclei in organoids, mean organoid sizes and the total amount of organoids per 3,872,215 μm^2^ area (49 fields of view) measured in individual wells at 15 different heights. PC346C PDXOs were processed and exposed to a dose range of docetaxel using the optimized procedure (mean +/− SD of five technical replicates per condition). The dashed line represents the mean before treatment initiation (day 0). Tumor growth on day 10 was determined for each indicated treatment dose by normalizing to day 0 controls and was subsequently compared to tumor growth on day 10 for untreated controls using an unpaired *t*-test. * *p* < 0.01; ns: not significant. (**B**) Representative confocal images (maximum projection) and image-based quantification of Hoechst 33342-stained PC346C PDXOs processed and exposed to a dose range of enzalutamide using the optimized procedure (mean +/− SD of five technical replicates per condition). Scale bar: 50 μm. Total amount of cell nuclei in organoids, mean organoid sizes and total amount of organoids per 4,972,068 μm^2^ area (63 fields of view) measured in individual wells at 15 different heights. Unpaired *t*-test for day 10 untreated control versus indicated treatment dose, which were both normalized to day 0; * *p* < 0.01; ns: not significant.

The sampled number of cell nuclei in organoids, organoid sizes and the number of organoids were quantified as direct measures of organoid load. When PC346C PCa PDXOs were exposed to docetaxel and enzalutamide, dose-related decreases in organoid sizes and culture density were detected (Figure 4A,B), confirming our brightfield observations in Figure 2D. A comparison with the growth of untreated day 10 controls confirmed tumor growth inhibition, with significant growth differences at the highest doses. 

**Figure 5:**
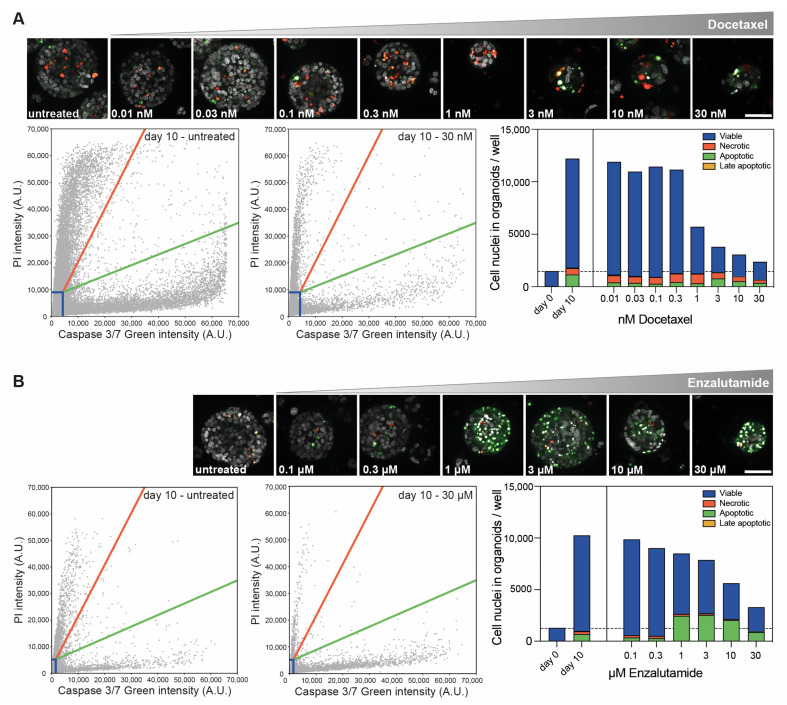
Image-based quantification of cell death in PCa PDXOs exposed to standard-of-care therapeutics. (**A**) Representative confocal images (maximum projections) of PC346C PDXOs exposed to a dose range of docetaxel for 10 days using the optimized procedure and stained with a dye combination of Hoechst 33342, Caspase 3/7 Green and PI. Scale bar equals 50 μm. Scatterplots of nuclear Caspase 3/7 Green and PI intensity in untreated controls and PDXOs exposed to 30 nM docetaxel. Gating thresholds were determined on day 0 and day 10 untreated controls (see Materials and Methods Section and Supplementary Figure S1). Bar chart of cell death quantification based on gating in corresponding scatterplots (mean of five technical replicates per condition). Dashed line represents the mean number of cell nuclei in organoids before treatment initiation (day 0). (**B**) Representative confocal images (maximum projections), scatter plots and cell death bar chart of PC346C PDXOs exposed to a dose range of enzalutamide for 10 days and stained with a dye combination of Hoechst 33342, Caspase 3/7 Green and PI. Scale bar: 50 μm.

In addition, confocal imaging revealed distinct cellular responses to both drugs. Ten days of exposure to docetaxel (0.01–30 nM) did not elicit cell death (Figure 5A), but it did inhibit organoid growth (Figure 4A), supporting docetaxel’s cytostatic capacities. In contrast, 10-day enzalutamide exposure induced apoptotic cell death at a dose of 1 μM and higher (Figure 5B), while exerting a growth-inhibitory effect only at the highest doses (10 and 30 μM, Figure 4B). 

For this imaging-based procedure, the same standard quality requirements were imposed as those described for the viability-based procedure: CV < 0.22, Z-factor > 0.4 and, where applicable, DMSO effect between 0.8 and 1.2. In addition, we included controls to validate cell death identification by exposing untreated organoid replicates to staurosporine for six hours prior to imaging (Supplementary Figure S7).

## 4. Discussion

There is an urgent need for standard operating procedures for organoid drug testing, which would allow better comparisons to be made between data from different research groups [12]. To this end, we developed an optimized procedure for processing and treating preassembled PCa PDXOs. Commonly used viability assays were complemented with essential quality checks, and a newly designed live-cell imaging pipeline was generated, offering additional insights into the mechanistic actions of the tested drugs. With minimal adaptions, these methods can be applied in tumor organoids derived from other cancer types to pursue the ultimate goal of increasing the organoid-based drug test validity and accelerate the clinical implementation of tumor organoids in oncology precision medicine.

We obtained highly consistent drug testing results using our methods, provided that a few quality standards had been met. We adhered to common quality checks (such as the CV, Z-factor and DMSO effect), but we also emphasize the importance of actively proliferating organoids during drug-exposure experiments, making baseline measurements (before treatment initiation) an indispensable component of the procedure. Specifically in PCa PDXOs, we found Y-27632 to be essential for assuring organoid proliferation. These findings are in concordance with the increased proliferation efficiency observed in benign prostate epithelial cells upon exposure to Y-27632, both in 2D and in 3D [24].

Previously, we described the impact of using preassembled organoids versus single organoid cells for drug testing [21]. Inherent to this strategy is the presence of organoid heterogeneity, both between and within technical replicates. Well-to-well heterogeneity in organoid size has been described as a major factor introducing variation in whole-well viability read-outs [13,25]. To reduce test result variability between wells (and comply with the CV and Z-factor requirements), we incorporated two interventions that prevent large variations in organoid size distribution: preassembled organoids are filtered to exclude organoids > 100 μm in diameter at the start of the experiment, and the total experiment duration is restricted to the minimal timeframe required for capturing drug response. 

Besides size variation, individual organoids can also vary in terms of drug response [26]. These intra-well differences between organoids cannot be determined with bulk read-outs, such as cell viability. Imaging, on the other hand, provides opportunities for single-organoid analysis or even individual organoid tracking over time [16,17,18,27,28,29,30]. Moreover, multiple parameters or biological endpoints can be evaluated at the same time [31]. For example, brightfield imaging has been employed to study multiple morphometric changes during or after organoid drug exposure, such as organoid size, shape and structure [28,29,32,33].

This approach also allows the monitoring of organoid growth and has been combined with fluorescent cell death markers to distinguish cytostatic from cytotoxic drug effects [17,33]. Often, a single cell death marker is used to identify dead organoid cells [16,17,18,33]. However, in our imaging-based procedure, we aimed to capture both organoid growth and a range of cell death modalities, including apoptosis and necrosis, within each individual organoid. For that reason, we multiplexed Hoechst 33342 nuclear dye with fluorescent cell death dyes, Caspase 3/7 Green and propidium iodide, which exhibit distinct functionalities and distinct emission spectra. We specifically opted for a minimalistic approach with the addition of dyes (avoiding immunolabeling, transfections or transductions), which makes rapid screening within clinically relevant timeframes feasible. The use of fluorescent protein-labeled organoids for evaluating organoid drug response has been successfully applied by others [16], but it requires longer experimental timelines, comes with varying transfection/transduction efficiencies and, most importantly, risks losing tumor heterogeneity.

Besides the well-known microscopy challenges to using 3D cell culture models, such as light absorption and scattering within the tissue, a few aspects inherent to our imaging procedure and image analysis pipeline need attention. We imaged a selected number of fields and planes per well in order to constrain the amount of data generated, at the cost of potential sampling bias. With our sampling approach, we may not always capture the largest intersection of each imaged organoid, and a part of an organoid can be missed at the edge of an imaging field. Both of these result in an underestimation of the organoid size, potentially falsely excluding organoids below the size threshold (area < 250 μm^2^). Conversely, for large organoids, multiple intersections are captured and counted as individual organoids, thus overestimating the total organoid number. Therefore, the parameters expressed represent the sampled number of organoids, organoid sizes and number of cell nuclei in organoids, without making the claim they are the absolute measurements of the entire wells. Whole-well detailed Z-stacks with small step sizes and 3D image analysis can solve these issues, but also drastically prolong the imaging time and greatly enlarge the data volumes when they are applied in large-scale drug screening [31]. The same concern arises when extensive time-lapse imaging is performed. The latter one would also induce a considerable amount of phototoxicity, which might interfere with correct interpretation of the drug’s effect [5,34]. For those reasons, and in light of future large-scale drug screen applications, we opted for endpoint measurements instead of live organoid tracking at multiple time points.

The rate of organoid cell death might be slightly underestimated in our procedure due to the loss of Hoechst signal and segmentation difficulties in multi-fragmented nuclei. Nevertheless, cytostatic versus cytotoxic effects were clearly distinguished. The number of dead cells remained stable upon the use of higher doses of docetaxel, while the percentage of dead cells increased due to a reduction in the total cell amount, indicating a growth-inhibitory, cytostatic effect. Enzalutamide, on the contrary, induced cytotoxicity upon the use of a higher treatment dose, with increasing dead cell counts. The relatively high number of dead organoid cells observed on day 10 for untreated controls can be attributed to organoids becoming larger, as has also been described by others [16].

As multiple individual organoids are imaged in each well, this imaging-based procedure has the advantage that every sampled organoid can be considered as a technical replicate [5,28]. This greatly increases the total number of replicates, and thus, the robustness of screening results as compared to that of viability assays, which typically rely on from three to six (whole-well) technical replicates per condition. Moreover, the applied Hoechst 33342 nuclear stain allows the verification of potential murine contamination of PDXO cultures [3,35,36], without the need for an additional work-up. Finally, this live-organoid imaging-based approach holds great promise for future drug testing in more complex in vitro 3D models that integrate multiple cell types. Individual cell types and their unique drug responses could be distilled to provide further insights in global tumor drug responses, including the role of the tumor micro-environment. 

## 5. Conclusions

We provide an optimized preclinical procedure for the processing of PCa PDXOs for medium-throughput drug testing, which can be adapted for other types of patient- and PDX-derived tumor organoids. Essential elements involve the use of preassembled organoids, the continuous presence of Y-27632 in order to ensure organoid proliferation, an extended drug exposure time and, finally, the verification of dedicated quality control metrics in each experiment. With this procedure we aim to improve the validity of currently applied bulk drug testing methods and allow the better comparison of data across studies. Moreover, we present a newly developed live-organoid imaging pipeline that offers opportunities for investigating drug-induced cellular responses (cytostatic versus cytotoxic) at the single-organoid level in large-scale oncology drug testing and for exploring individual organoid drug response. Applying this imaging pipeline in early passages of primary PDOs would allow early and rapid drug testing with a minimal loss of intra-patient tumor heterogeneity, making it highly attractive for personalized treatment selection.

## Data Availability

All data generated in this study are available from the corresponding author on reasonable request. PDXOs are available from W.M.v.W. under a material transfer agreement with the Erasmus MC.

## Supplementary Materials

The following supporting information can be downloaded at: https://www.mdpi.com/article/10.3390/cells12101377/s1, Figure S1: Gating thresholds for cell death quantification; Figure S2: Optimization of PDXO drug testing conditions; related to Figure 1E; Figure S3: Impact of drug exposure time on variability; related to Figure 1F; Figure S4: Reproducibility of the optimized viability-based procedure; related to Figure 2; Figure S5: Variability in organoid proliferation; Figure S6: Verification of spectral crosstalk between fluorescent dyes Caspase 3/7 Green and propidium iodide; Figure S7: Staurosporine exposure as positive control for cell death induction; Table S1. Composition of adjusted prostate cancer organoid medium (APCOM) and basic prostate growth medium (PGM basic). References [37,38] are cited in Supplementary Materials.
