# Supplementary material for: Viability Analysis and High-Content Live-Cell Imaging for Drug Testing in Prostate Cancer Xenograft-Derived Organoids
Source: Cells. 2023 May 12;12(10):1377. doi: 10.3390/cells12101377 (PMC10216787; doi:10.3390/cells12101377)
Supplement: Supplementary file 1 [file cells-12-01377-s001.zip › cells-2386019-supplementary.pdf]

Figure S1

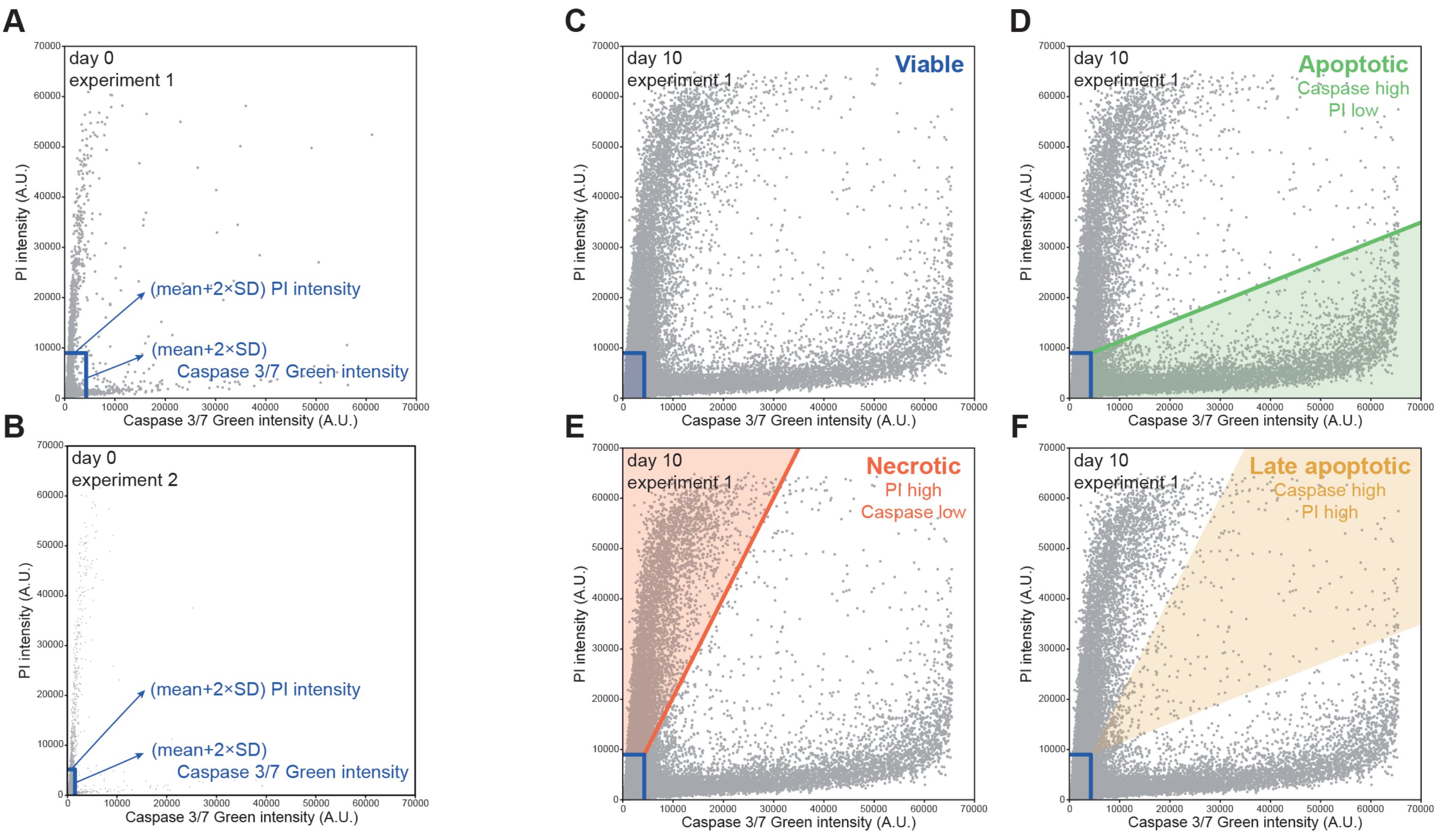

Figure S1. Gating thresholds for cell death quatification. (A-B) Scatterplots of nuclear Caspase 3/7 Green and PI intensity of day 0 PC346C PDXOs, before exposure to docetaxel (A, experiment 1) and before exposure to enzalutamide (B, experiment 2). Each dot represents one organoid nucleus. **(C-F)** Gating thresholds for viable cells (blue), apoptotic cells (green), necrotic cells (red) and cells in late apoptosis or secondary necrosis (orange) in untreated day 10 PC346C PDXOs, in correspondence to (A).

Figure S2

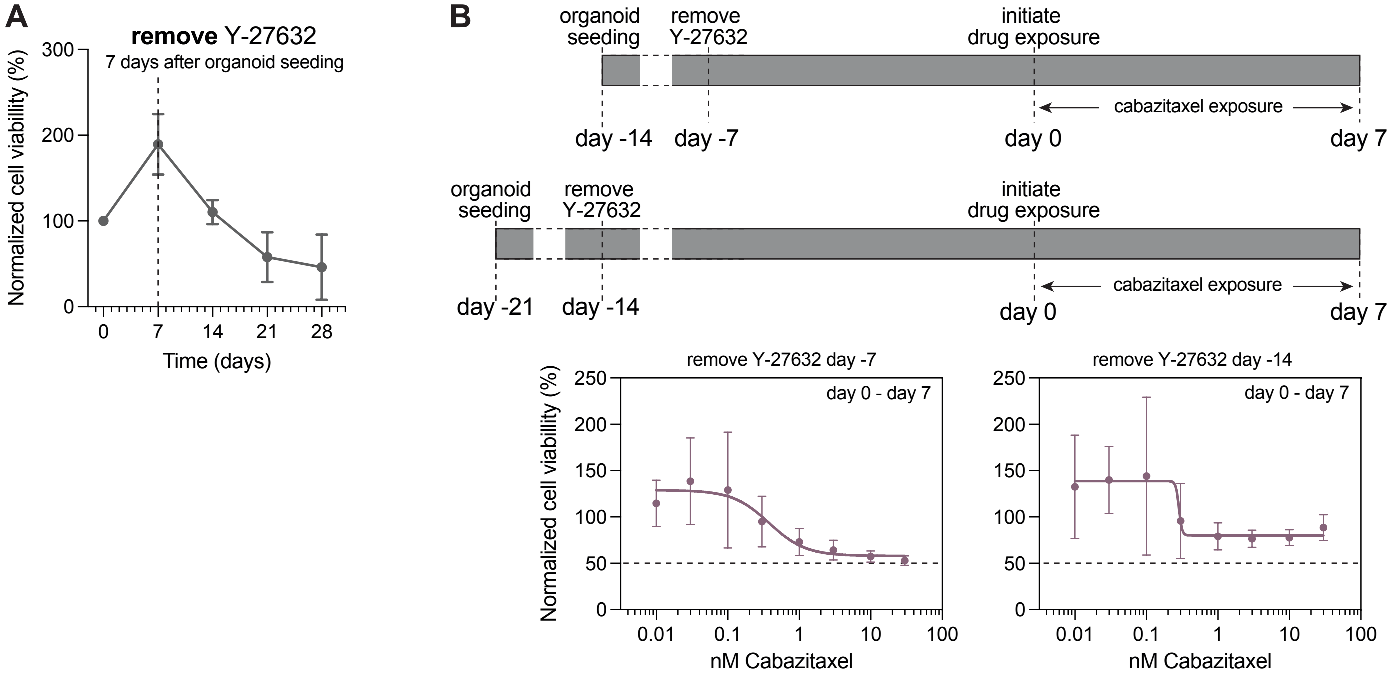

Figure S2. Optimization of PDXO drug-testing conditions, related to Figure 1E. (A) Impact of Y-27632 on organoid proliferation. Viability of PC2412 PDXOs from the day of seeding, with Y-27632 removed from organoid culture medium after 7 days. **(B) Impact of organoid viability on treatment efficiency,** in correspondence to (A). Y-27632 was removed from culture medium 7 days after organoid seeding and 7 to 14 days prior to drug exposure, as indicated on the timelines. PC2412 PDXOs were exposed to cabazitaxel for 7 days (mean +/- SD of six technical replicates per dose). Normalization to vehicle (ethanol) controls.

Figure S3

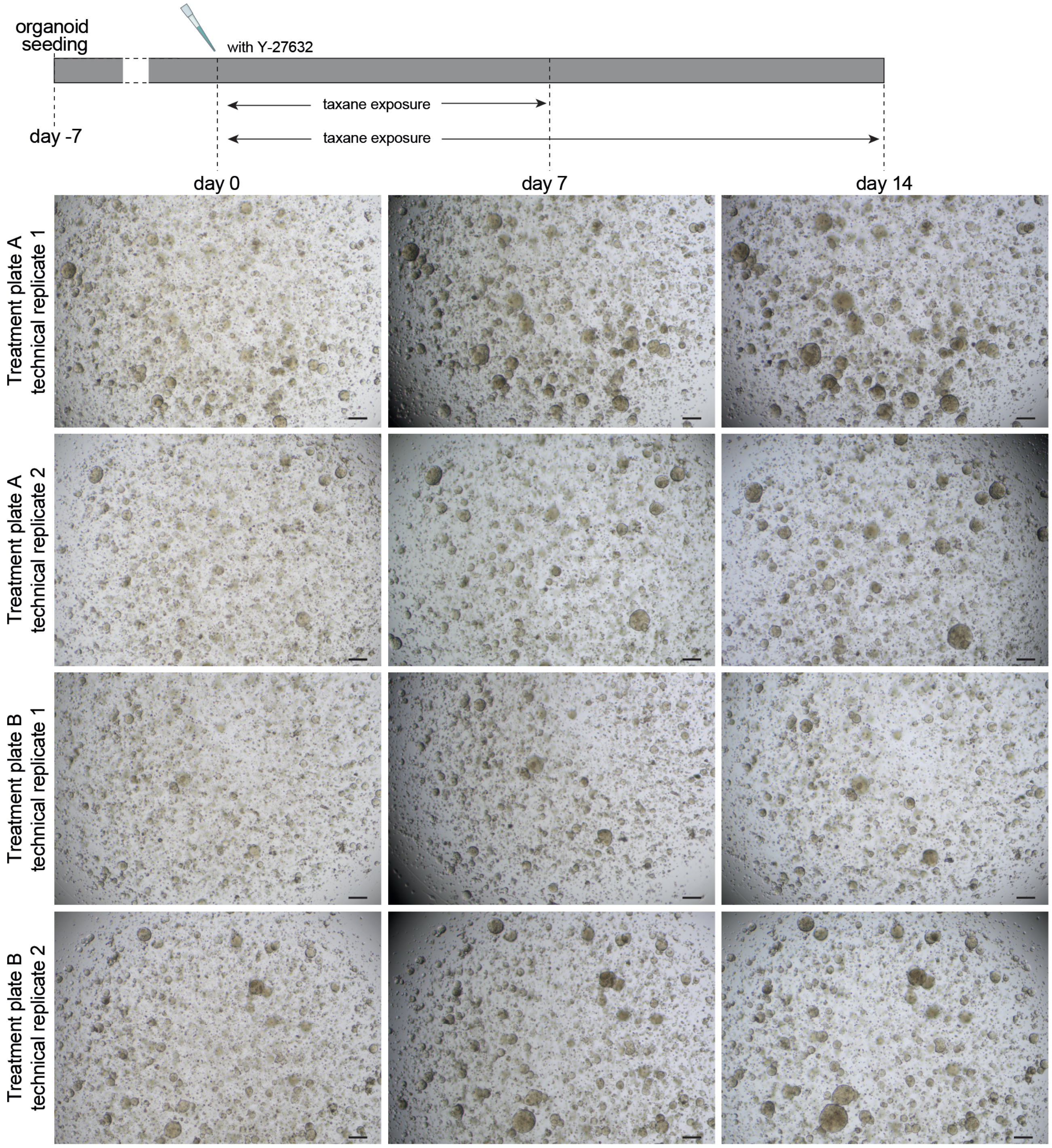

Figure S3. Impact of drug exposure time on variability, related to Figure 1F. Representative brightfield images of PC2416-DEC PDXO untreated controls at day 0, day 7 and day 14 after treatment initiation, as indicated on the timeline. Images are shown for two different wells (technical replicates) on two different treatment plates (plate A and B). Scale bars equal 100 µm.

Figure S4

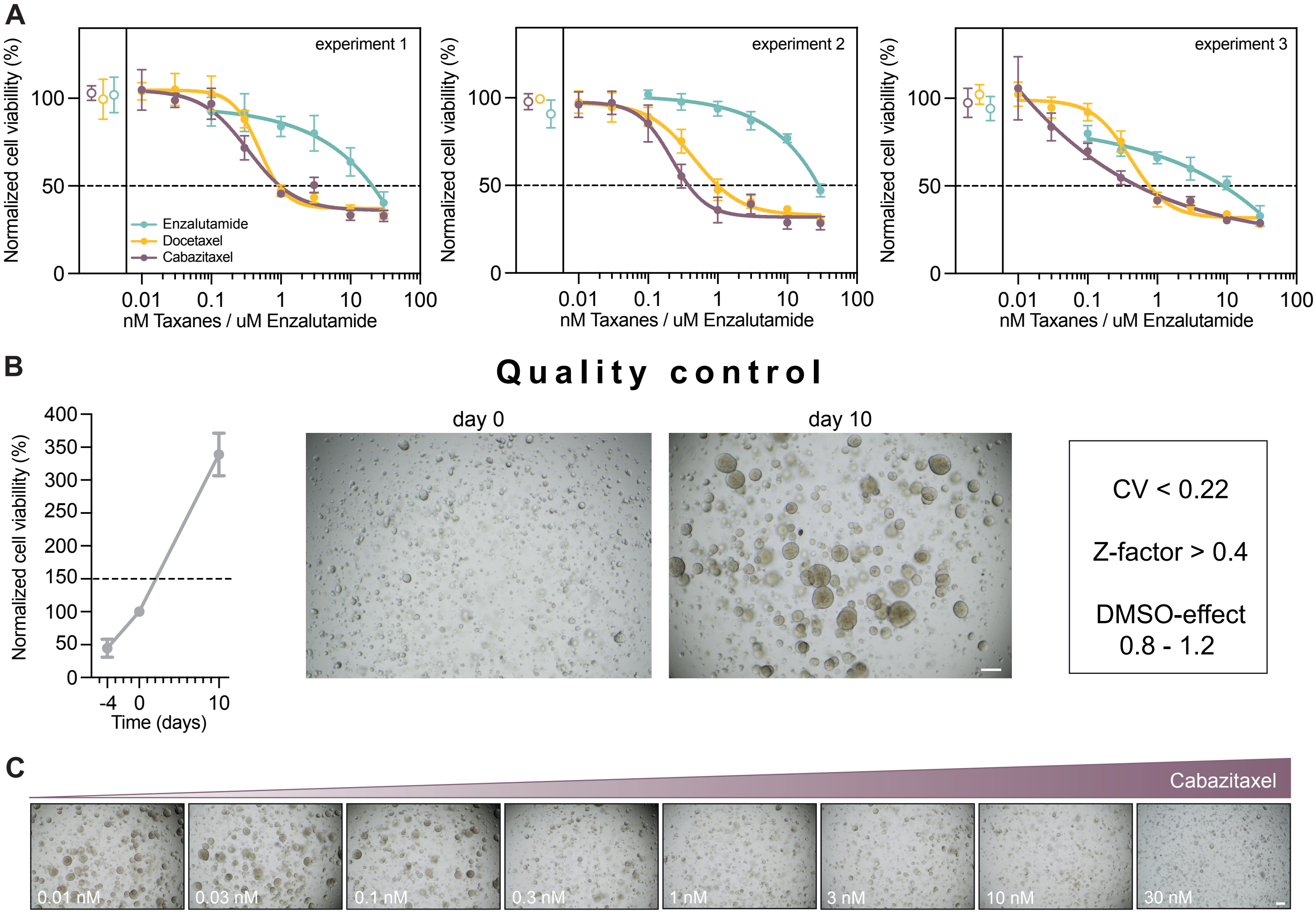

Figure S4. Reproducibility of the optimized viability-based procedure, related to Figure 2. (A) Three independent experiments of PC346C PDXOs exposed to dose-ranges of enzalutamide, docetaxel and cabazitaxel (mean +/- SD of six technical replicates per condition). Normalization to all untreated controls per experiment. Open dots represent untreated controls of each treatment plate. **(B)** Quality control metrics of experiments in (A): i. Increase in organoid viability of at least 1.5 between day 0 and day 10 (mean +/- SEM of three experiments, 6 to 18 technical replicates for each timepoint) with representative brightfield images of day 0 and day 10 untreated controls, scale bar 100 µm; ii. Coefficient of variation (CV) < 0.22 and Z-factor > 0.4 for each treatment plate; iii. DMSO-effect between 0.8 and 1.2 for each enzalutamide treatment plate. **(C)** Representative brightfield images of PC346C PDXOs exposed to a dose-range of cabazitaxel. Scale bar 100 µm.

Figure S5

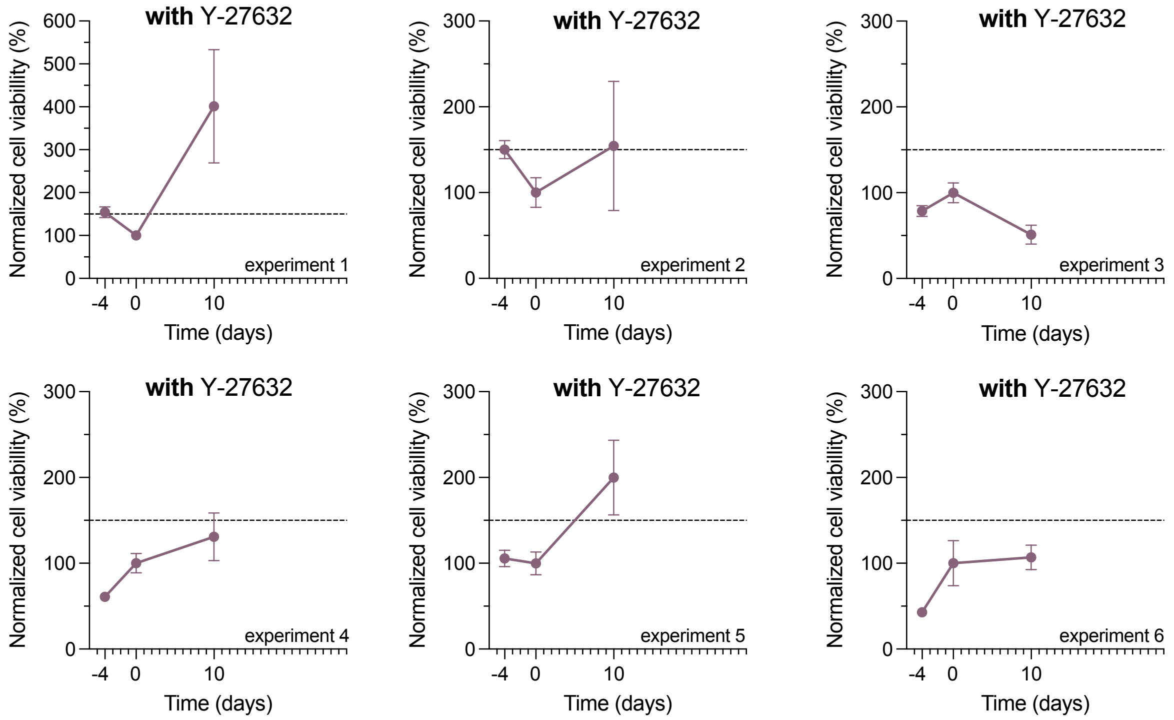

Figure S5. Variability in organoid proliferation. Viability of PC2412 PDXOs from the day of seeding, with Y-27632 continuously present in organoid culture medium. Six independent experiments. Mean +/- SD of 6 to 18 technical replicates. Dashed line indicates threshold of 1.5 increase in organoid viability between day 0 and day 10.

Figure S6

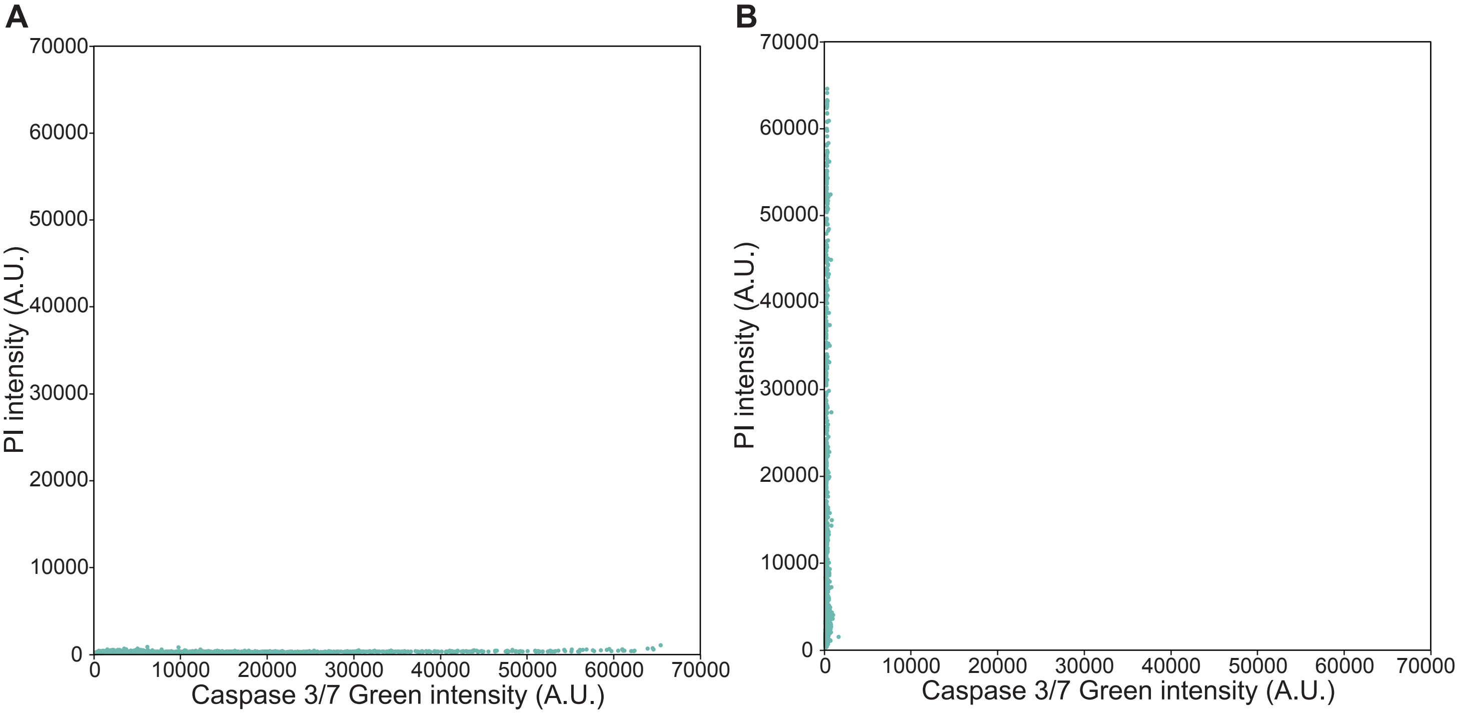

Figure S6. Verification of spectral crosstalk between fluorescent dyes Caspase 3/7 Green and PI. Scatterplots of nuclear Caspase 3/7 Green and PI intensity of day 0 PC346C PDXOs exposed to 1 μM staurosporine six hours before imaging. Each dot represents one organoid nucleus. PDXOs were stained with either Caspase 3/7 Green and Hoechst **(A)** or PI and Hoechst **(B)**.

Figure S7

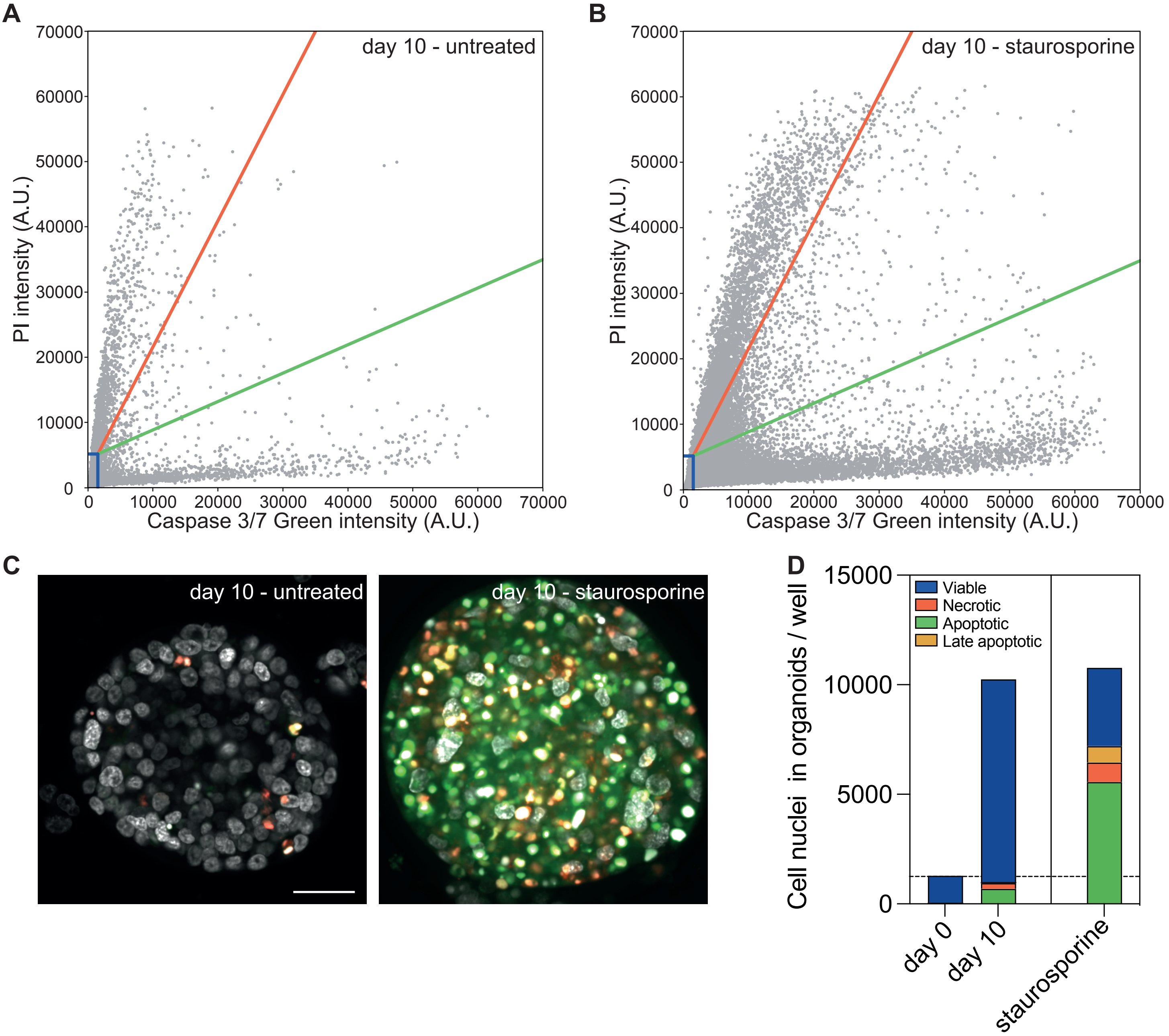

Figure S7. Staurosporine exposure as positive control for cell death induction. (A-B) Scatterplots of nuclear Caspase 3/7 Green and PI intensity of day 10 PC346C PDXOs: (A) untreated, (B) exposed to 1µM staurosporine six hours before imaging. Each dot represents one organoid nucleus. Gating thresholds were determined on day 0 (Supplementary Figure S1B) and day 10 untreated controls. **(C)** Representative confocal images of untreated and staurosporine-treated PC346C PDXOs. Scale bar equals 50µm. **(D)** Bar chart of cell death quantification, based on gating in corresponding scatterplots (mean of five technical replicates per condition). Dashed line represents the mean number of organoid nuclei before treatment initiation (day 0).

Table S1. Composition of Adjusted Prostate Cancer Organoid Medium (APCOM) and basic Prostate Growth Medium (PGM basic).

MEDIUM	MEDIUM COMPONENTS	SUPPLIER (catalogue number)	CONCENTRATION
**APCOM** [3,20]	Advanced DMEM/F12 (AdDMEM/F12)	Thermo Fisher Scientific, Waltham, Massachusetts, USA (cat. no. 12634010)	-
	Hepes	ThermoFisher Scientific (cat. no. 15630056)	10 mM
	L-Glutamine	Lonza (cat. no. BE17-605E)	2 mM
	Penicillin/Streptomycin	Lonza (cat. no. DE17-602E)	100 U/mL
	Epidermal Growth Factor (EGF)	Sigma-Aldrich, Saint Louis, Missouri, USA (cat. no. E9644)	20 ng/mL
	Fibroblast Growth Factor 2 (FGF-2)	R&D Systems, Minneapolis, Minnesota, USA (cat. no. 233-FB-025)	5 ng/mL
	Fibroblast Growth Factor 10 (FGF-10)	PeproTech, Cranbury, New Jersey, USA (cat. no. 100-26)	10 ng/mL
	Noggin	Conditioned medium from Hek293T-Noggin-Fc [37]	-
	R-Spondin	Conditioned media from Hek293T-hRSpo1 [38]	-
	A 83-01	Tocris Bioscience, Abingdon, UK (cat. no. 2939)	500 nM
	Prostaglandin E2 (PGE2)	Tocris Bioscience (cat. no. 2296)	1 µM
	R1881	Sigma-Aldrich (cat. no. R0908)	0.1 nM
	Y-27632 dihydrochloride	Adipogen, San Diego, California, USA (cat. no. AG-CR1-3564-M025)	10 µM
**PGM basic** [19]	DMEM/F12	Lonza, Basel, Switzerland (cat. no. BE12-719F)	-
	Penicillin/Streptomycin	Lonza (cat. no. DE17-602E)	100 U/mL
	Epidermal Growth Factor (EGF)	Sigma-Aldrich, Saint Louis, Missouri, USA (cat. no. E9644)	10 ng/mL
	Insulin-Transferrin-Selenium (ITS) (100 X)	ThermoFisher Scientific (cat. no. 41400045)	1 X
	Bovine Serum Albumin Fraction V (BSA)	Roche Diagnostics, Mannheim, Germany (cat. no. 10735094001)	0.01% (w/v)
	FBS	ThermoFisher Scientific (cat. no.10270106)	2% (v/v)
